# From Mechanisms to Practice: Gut Microbiome-Based Strategies for Supporting Recovery in Elite Athletes

**DOI:** 10.3390/nu18142403

**Published:** 2026-07-22

**Authors:** Junior Carlone, Paolo Sgrò, Attilio Parisi, Alessio Fasano

**Affiliations:** 1 Department of Neurosciences, Biomedicine and Movement, University of Verona, 37134 Verona, Italy; 2 Department of Movement, Human and Health Sciences, University of Rome “Foro Italico”, 00135 Rome, Italy; 3 Division of Pediatric Gastroenterology and Nutrition, Mass General for Children and Harvard Medical School, Boston, MA 02114, USA; 4 Department of Nutrition, Harvard T.H. Chan School of Public Health, Boston, MA 02115, USA; 5 European Biomedical Research Institute of Salerno, 84131 Salerno, Italy

**Keywords:** gut microbiome, athletic recovery, probiotics, short-chain fatty acids, exercise-induced inflammation

## Abstract

Recovery in elite athletes represents a critical determinant of performance and health outcomes. The gut microbiota has been proposed as a modulating factor for recovery through anti-inflammatory mechanisms, oxidative stress management, sleep regulation, and biosynthetic potential for essential micronutrients. This review examines the mechanisms linking gut microbiota composition and function to athletic recovery and critically evaluates the evidence supporting its application in sports medicine. Athletes appear to harbor a more enriched microbial biosynthetic potential, with substantially greater numbers of high-biological-impact synthases involved in the production of vitamins, amino acids, and bioactive metabolites. Short-chain fatty acids, particularly butyrate and propionate, have demonstrated anti-inflammatory effects in preclinical studies, with emerging evidence in humans. The gut–brain axis has been proposed to modulate recovery by regulating neurotransmitter production and controlling circadian rhythms. Sport-associated microbial signatures seem to reflect metabolic demands, with endurance athletes showing enrichment for *Prevotella* and *Veillonella*, while strength athletes tend to harbor higher levels of proteolytic bacteria. Probiotic interventions with multi-strain *Lactobacillus* and *Bifidobacterium* formulations have reported reductions in inflammatory markers, improvements in oxidative stress biomarkers, and enhanced sleep quality in small-scale randomized controlled trials involving athletic populations, and improvements in self-reported sleep quality in a controlled, non-randomized study in elite athletes. Optimizing gut microbiota composition and function offers a promising complementary strategy for enhancing recovery in elite athletes. Potential applications that require prospective validation include sport-specific probiotic interventions, nutritional strategies to enhance short-chain fatty acid production, and the integration of microbiota assessment with traditional recovery monitoring. Further research is needed to establish standardized protocols and identify predictive biomarkers of individual response to microbiota-targeted interventions.

## 1. Introduction

In elite sport, strategies to support recovery are expanding beyond established physical and nutritional modalities, and among the most novel is modulation of the gut microbiota, a system whose role in metabolism and immunity is well characterized but whose contribution to athletic recovery remains comparatively underexplored at the intervention level. Recovery after intense physical exercise constitutes one of the most critical factors for maintaining and optimizing physical performance in elite athletes [1]. This physiological stress induces a series of acute responses and longer-term adaptations, including increased oxidative stress (OS), intestinal permeability, exercise-induced muscle damage, a systemic inflammatory response, and modulation of hormonal responses [2,3,4,5,6,7]. These adaptations, if not adequately managed, can prolong recovery times and alter subsequent performance, compromising the athlete’s health. Traditionally, recovery strategies have focused on physical and nutritional modalities, including active and passive rest, cold baths, cryotherapy, manual or compressive therapies, and nutraceutical and pharmacological approaches [1]. However, the growing understanding of the role of the gut microbiota in human physiology has opened new perspectives for optimizing athletic recovery [8].

The gut microbiota has emerged as a multifunctional system capable of influencing human physiology well beyond digestive function, with established roles in energy metabolism, immune regulation, neurotransmitter synthesis, and modulation of systemic inflammation [8,9,10,11]. In athletes, this microbial ecosystem, which may itself function as an endocrine organ, displays distinct features compared with sedentary individuals, characterized by greater microbial diversity and specific taxonomic signatures associated with athletic performance [12,13]. Physical exercise and gut microbiota exhibit a bidirectional relationship. Training and competition modulate microbial composition, while the microbiota can influence recovery processes through the production of bioactive metabolites, modulation of inflammatory response, and optimization of autonomic nervous system function [14,15]. This bidirectionality positions the gut microbiome as a potential target for optimizing athletic recovery. Although it has been increasingly characterized at the descriptive level, the functional significance of microbiome modulation in elite athletes remains unclear. While training and diet are established modulators of the gut microbiota, whether microbiome alterations directly contribute to recovery and performance adaptations or merely reflect physiological responses to exercise remains unresolved. Furthermore, current evidence is predominantly correlational.

This narrative review examines and critically appraises the current evidence on gut microbiome-based strategies for optimizing recovery in elite athletes. Specifically, it addresses the mechanistic pathways through which the gut microbiome modulates key recovery processes, sport-specific microbiome adaptations relevant to recovery, and potential practical applications for sports medicine practitioners.

## 2. Methodological Approach

This narrative review was conducted without a pre-registered protocol or systematic data extraction, and the selection and interpretation of the literature reflect the authors’ judgment and expertise. The conduct and reporting of the review were guided by the Scale for the Assessment of Narrative Review Articles (SANRA) (File S1) [16]. The literature was identified through searches of PubMed, Scopus, and Web of Science, with the search period extending up to July 2026. Search terms were combined as follows: (“gut microbiome” OR “gut microbiota” OR “probiotics” OR “prebiotics”) AND (“athlete” OR “exercise” OR “competition”) AND (“recovery” OR “inflammation” OR “oxidative stress” OR “short-chain fatty acids” OR “sleep”). Searches were complemented by manual screening of reference lists and the authors’ prior knowledge of the field.

Evidence was appraised with priority given to systematic reviews, meta-analyses, and randomized controlled trials involving athletic or physically active populations. Where athlete-specific evidence was insufficient, mechanistic studies and findings from non-athletic clinical populations were incorporated, provided that they were biologically plausible in the context of exercise and recovery, supported by consistent findings across different models or study designs, and not contradicted by athlete-specific evidence. Where such evidence is used to support a specific claim, its preclinical or non-athletic origin is explicitly indicated in the text. Practical recommendations are informed primarily by athlete-specific evidence, with complementary support from non-athletic studies where appropriate.

As with all narrative reviews, this approach is subject to selection bias, and no formal count of identified versus included records was maintained. Findings should therefore be interpreted with consideration of the heterogeneity of study designs, populations, and outcome measures across the included literature.

## 3. Exercise-Induced Inflammation and Microbiome Modulation

### 3.1. Inflammatory Response to Exercise

Intense physical exercise induces a systemic inflammatory response characterized by a complex pattern of adaptations that involve both cellular and molecular components of the immune system [17]. This response functions as an essential adaptive physiological process that facilitates tissue repair and enhances performance. However, it can become problematic when the intensity, duration, or frequency of exercise exceeds the organism’s ability to recover [18]. During physical exercise, a significant increase in circulating cytokines occurs, including anti-inflammatory cytokines such as interleukin-6 (IL-6), which is pleiotropic, and pro-inflammatory cytokines such as tumor necrosis factor-alpha (TNF-α) and interleukin-1 beta (IL-1β) [19]. IL-6, in particular, can increase its levels after physical exercise, serving as a sensitive indicator of metabolic, mechanical, and energetic stress; for this reason, it is defined in this context as a myokine, a cytokine produced and released by contracting skeletal muscle [20]. Moreover, the characteristics of inflammation can vary by sport, leading to distinct and specific inflammatory patterns. The temporal dynamics of the inflammatory response can also follow predictable patterns through distinct phases, depending on the type of physical exercise [20,21]. Exercise-induced muscle damage functions as a principal trigger of the inflammatory response in athletes. This process is characterized by microlesions of muscle fibers and alterations in sarcolemma integrity [22]. The extent of muscle damage can vary depending on the type of exercise, with eccentric contractions inducing significantly greater increases in creatine kinase (CK) compared to concentric contractions [23,24]. Endurance sports can determine particularly marked inflammatory responses. In particular, IL-6, a key cytokine produced by skeletal muscle during prolonged aerobic activity, increases notably during a marathon, remaining elevated for up to 72 h post-race [21,25,26]. This response not only reflects tissue damage but also serves as a possible regulatory and adaptive mechanism, as IL-6 exerts anti-inflammatory effects by stimulating the production of interleukin-10 (IL-10) and interleukin-1 receptor antagonist (IL-1ra), and inhibiting TNF-α [27]. This illustrates the context-dependent, dual nature of IL-6: whereas chronically elevated IL-6 is a hallmark of low-grade systemic inflammation in metabolic and chronic disease, the transient IL-6 released by contracting muscle during exercise predominantly acts as an anti-inflammatory myokine [27]. Despite IL-6’s established role as a myokine, the contribution of the gut microbiota to exercise-induced modulation of IL-6 remains poorly characterized. However, endurance athletes are known to be more prone to upper respiratory tract infections (URTI) and gastrointestinal (GI) issues [28]. Strength and power sports can elicit less prolonged inflammatory responses, primarily due to mechanical microtrauma and local immune cell infiltration [25,29]. However, over the long term, these types of exercise induce clear systemic anti-inflammatory adaptations. A recent meta-analysis conducted in middle-aged and older adults, rather than in athletic populations, reported a significant reduction in C-reactive protein (CRP) following resistance exercise programs, without concomitant changes in TNF-α [30]. Team sports, finally, exhibit hybrid characteristics, combining high-intensity phases with periods of active recovery. In these sports, acute inflammatory responses are observed, characterized by an initial increase in IL-6 and TNF-α levels, followed by a subsequent increase in CRP on the morning following competition [31]. These patterns could reflect both metabolic and mechanical stress, as well as the physical contact typical of intermittent high-intensity disciplines. In this inflammatory context, the immune system of elite athletes is continuously stressed. With adequate training programming and periodization, this stress can promote adaptive immune responses; however, inadequate management may compromise immune function and prolong recovery times [18].

### 3.2. Gut Microbiome and Exercise-Induced Immune Modulation

In this context, the gut microbiota has been proposed as a potential modulator of exercise-induced inflammation, acting through the production of bioactive metabolites, the regulation of intestinal barrier integrity, and the interaction with both innate and adaptive immune components.

The gut microbiota and potential exogenous probiotic supplementations could play a central role in modulating immune response [32,33,34]. Microbiota modulates both innate and adaptive immunity, and short-chain fatty acids (SCFAs) can promote the differentiation of Regulatory T cells (Tregs) in humans, which are essential for containing excessive inflammation and maintaining immune homeostasis [35,36]. Clarke et al. observed that rugby players with high microbial diversity have positively correlated elevated CK levels, higher protein consumption, and a more favorable inflammatory and metabolic profile [12]. However, assuming a priori that physical exercise induces either positive or negative immune adaptations can be misleading. When appropriately managed, physical exercise can positively impact immune function [12,37]. Recent studies have confirmed that intense exercise, particularly in hot environments, can increase intestinal permeability, allowing bacterial endotoxins, such as lipopolysaccharide (LPS), to pass into the bloodstream [38,39,40]. A balanced microbiota, particularly the production of SCFAs, especially butyrate, contributes to maintaining intestinal barrier integrity. Murine models and in vitro studies on human cells have shown that butyrate can strengthen tight junctions (TJs) of the intestinal epithelium by activating Hypoxia-Inducible Factor-1 (HIF-1), while also promoting mucus production [41,42,43,44]. These effects may help reduce intestinal permeability. Against this background, the gut microbiota may contribute to modulating the systemic inflammatory response in athletes through multiple interaction pathways, potentially supporting the management of both acute exercise-induced OS driven by the significant increase in reactive oxygen species (ROS) production that accompanies intense physical exercise, and the chronic low-grade inflammatory state associated with intestinal dysbiosis, characterized by alterations in microbial composition and diversity, that may compromise recovery processes [3,45,46,47]. A eubiotic microbiota, defined as a balanced and diverse microbial community associated with host health, may exert anti-inflammatory effects through the production of bioactive metabolites and the maintenance of intestinal barrier integrity, whereas dysbiosis may contribute to impaired host homeostasis, highlighting the gut microbiota as an attractive target for optimizing athletic recovery [2,45,46].

Exercise-induced inflammation should not be interpreted exclusively as a pathological process, but rather as an essential physiological phase for muscular and systemic remodeling. The nature and intensity of the exercise determine the extent and quality of the inflammatory response, which functions as an essential mediator of adaptation and health in both the short and long term. Elite athletes exhibit distinct mechanisms for modulating their gut microbiota, leading to specific modifications in functional taxonomic profiles that facilitate SCFAs production [48,49]. Beyond these established mechanisms, recent in vivo and in vitro studies have opened new perspectives that consider tryptophan metabolism pathways. Indolic derivatives, including primarily indolepropionic acid (IPA), indolelactic acid (ILA), indoleacetic acid (IAA), indole acrylate (IAC), and indole aldehyde (IAID), are produced through intestinal microorganism metabolism. These compounds may contribute to tryptophan metabolism, acting partly through the kynurenine (Kyn) pathway and exerting systemic protective actions [50,51]. Indolic metabolites may play an essential role in maintaining intestinal homeostasis and systemic immunity, potentially influencing the onset and progression of various pathological conditions. These include inflammatory bowel disease, obesity, metabolic syndrome, tumors, nervous system diseases, infectious diseases, cardiovascular diseases, and hepatic fibrosis [50]. Dietary fibers, beyond the established SCFAs production, appear to have a central role in tryptophan metabolism. Through multiple mechanisms, these may promote the differentiation and function of anti-inflammatory immune cells, including anti-inflammatory macrophages, Treg cells, CD4+ T helper cells, and CD8αα+ regulatory T cells, IL-10 and interleukin-35 (IL-35) regulatory B cells, and group 3 innate lymphoid cells (ILC3) producing interleukin-22 (IL-22), involved in maintaining intestinal mucosal homeostasis [50,51]. Furthermore, indolic metabolites exert their action by activating the aryl hydrocarbon receptor (AHR), thereby improving intestinal epithelial barrier integrity [52]. AHR is a transcription factor widely expressed by immune cells, and several studies have demonstrated that its activation modulates both innate and adaptive immune responses in a ligand-specific manner [52].

Additionally, bile acids (BAs) are important bile components that regulate immunity and inflammation [53]. These are classified into primary acids, such as cholic acid (CA) and chenodeoxycholic acid (CDCA), metabolized in the liver, and secondary acids, such as deoxycholic acid (DCA) and lithocholic acid (LCA), which become substrates for microbial metabolism in the colon through bacteria equipped with bile salt hydrolase (BSH) and 7α-dehydroxylase (7α-DH) [54]. BAs act as mediators, components, and effectors of the microbiota-bile acid-skeletal muscle axis, which may specifically regulate the activation of the NOD-like receptor family, pyrin domain-containing 3 (NLRP3) inflammasome through secondary BAs. This creates an exercise-mediated inflammatory control system that increases the abundance of bacteria associated with BAs production, activating signaling pathways mediated by farnesoid X receptor (FXR) and Takeda G protein-coupled receptor 5 (TGR5) [55,56].

These gastrointestinal tract mechanisms, including metabolic and signaling functions, are accompanied by specific morphological adaptations that show plasticity in response to external stimuli. Physical exercise has been associated with improved intestinal barrier integrity, mediated by zonulin modulation and increased expression of junctional proteins, such as zonula occludens-1 (ZO-1) and occludin [10,11,57]. The morphological response of the gastrointestinal tract to physical exercise follows a specific temporal dynamic: acute exposure may initially lead to increased intestinal permeability, partly due to elevated body temperature and the transient disassembly of TJs. However, progressive adaptation to structured physical exercise regimens can lead to compensatory strengthening of the intestinal barrier, thereby improving function [58].

**Figure 1:**
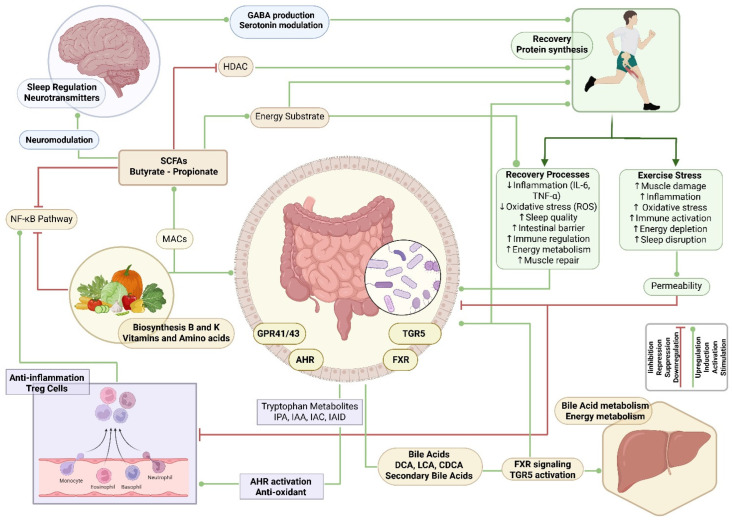
Gut microbiome-mediated recovery mechanisms in elite athletes. Integrated pathways showing how gut microbiota influences athletic recovery through: SCFAs-mediated anti-inflammatory effects and intestinal barrier enhancement, tryptophan-derived AHR activation and Tregs differentiation, bile acid signaling via FXR/TGR5, and enhanced vitamin and amino acid biosynthesis. (The figure provides an integrative mechanistic framework rather than definitive pathways established in athletes). (↑ = Increased; ↓ = Decreased; AHR = Aryl Hydrocarbon Receptor; CDCA = Chenodeoxycholic Acid; DCA = Deoxycholic Acid; FXR = Farnesoid X Receptor; GABA = Gamma-Aminobutyric Acid; GPR41/43 = G protein-coupled receptor 41/43; HDAC = Histone deacetylase; IAID = Indole-3-aldehyde; IAC = Indole-3-acrylic Acid; IAA = Indole-3-acetic Acid; IPA = Indole-3-propionic Acid; LCA = Lithocholic Acid; MACs = Microbiota-Accessible Carbohydrates; NF-κB = Nuclear Factor kappa-light-chain-enhancer of activated B cells; ROS = Reactive Oxygen Species; SCFAs = Short-Chain Fatty Acids; TGR5 = Takeda G protein-coupled Receptor 5; TNF-α = Tumor Necrosis Factor alpha; Tregs = Regulatory T cells; IL-6 = Interleukin 6).

Exercise-induced inflammation should be viewed as a context-dependent process requiring optimization rather than suppression. Sport-specific inflammatory signatures suggest that microbiota-targeted recovery strategies must be tailored to the metabolic demands of each discipline. The microbiota’s capacity to modulate this response through SCFAs production and maintenance of intestinal barrier integrity positions it as a key target for personalized recovery protocols (Figure 1).

## 4. Microbial Pathways in Recovery

### 4.1. Oxidative Stress Management

OS is a significant factor in determining physical performance and athlete health. Physical exercise, through muscle contraction, can induce ROS production in different tissues, accelerate muscle fatigue, and activate biochemical signaling pathways that contribute to exercise-induced adaptation in contracting muscle fibers [59]. These adaptations vary depending on the athlete’s training status and hormetic state, leading to either positive or negative adaptations that contribute to muscle damage and inflammation [60,61]. The effects of ROS production are dose-dependent, as observed with pro- and anti-inflammatory mechanisms mediated by cytokines, T cell populations, or responses to bacterial components in the intestine. Regulated ROS production performs vital functions, as it helps defend against pathogens and stimulate repair mechanisms. In contrast, excessive ROS levels, in the context of chronic inflammation, can cause tissue damage if not counteracted by antioxidant mechanisms [62].

The gut microbiota can contribute to OS management through several mechanisms, although the supporting evidence derives from non-athletic populations [63]. Some intestinal bacterial species can produce endogenous antioxidants or metabolize diet-derived antioxidant compounds, increasing their bioavailability [64]. The conversion of dietary polyphenols by the microbiota into bioactive metabolites could enhance the host’s antioxidant defenses [65]. A comparative study of soccer players and a non-athlete population revealed that diets rich in natural antioxidants are associated with lower levels of oxidative damage markers and higher dietary antioxidant indices than in non-athlete controls [66]. Moreover, gut microbiota could influence the expression of endogenous antioxidant enzymes such as superoxide dismutase (SOD) and catalase (CAT). For example, butyrate supports the intestinal barrier, modulates inflammation, and reduces OS, making it a key metabolite for maintaining cellular homeostasis [67,68,69].

Commensal bacterial communities exert a key role in intestinal reduction-oxidation (REDOX) homeostasis. Orders, families, genera, and species such as *Bacteroidales*, *Clostridiales*, *Bifidobacteriaceae*, *Erysipelotrichaceae*, *Faecalibacterium prausnitzii*, and *Lactobacillus* spp. can contribute to an antioxidative environment. Conversely, *Enterobacteriaceae*, *Fusobacteriaceae*, *Gemellaceae*, *Neisseriaceae*, *Pasteurellaceae*, *Veillonellaceae*, *Lachnospiraceae*, *Ruminococcus torques*, *Ruminococcus gnavus*, *Campylobacter jejuni*, *Salmonella Enteritidis*, *Shigella*, *Staphylococcus*, *Yersinia*, *Listeria* spp., and *Clostridium perfringens* can contribute to a pro-oxidative environment [62]. Although it should be noted that several of these taxa, including *Veillonellaceae* and *Ruminococcus*, are also recognized as important producers of SCFAs such as propionate and acetate, highlighting the context-dependent and strain-specific nature of their functional roles in the gut ecosystem. This apparent contradiction reflects both taxonomic resolution and the surrounding ecological context. A single family or genus may include strains with divergent metabolic outputs, and the net redox contribution of a given taxon depends on substrate availability, local oxygen tension, and the composition of the wider community. Moreover, bacterial communities that contribute to a pro-oxidative environment also develop specific mechanisms to survive in it, thereby inducing ROS production by intestinal epithelial cells [62].

An innovative role of the microbiota concerns the bacterial transformation of tryptophan, which may generate antioxidant compounds through specialized metabolic pathways. These transformations appear to be particularly associated with bacteria belonging to *Clostridium*, *Bacteroides*, *Bifidobacterium*, *Lactobacillus*, and *Peptostreptococcus* [52]. IPA may possess scavenging action against hydroxyl radicals and protection against oxidative damage in various tissues [52]. IAC has been shown to promote intestinal epithelial barrier function and mitigate inflammatory responses in murine models, favoring goblet cell differentiation and mucus production, which is mediated by AHR activation [52]. Recent studies have highlighted that indole-induced AHR activation can represent a mechanism through which bacteria contribute to mucosal homeostasis. Roager and Licht reported that *Lactobacillus* spp. may regulate IL-22 mucosal homeostasis through AHR activation induced by IAID, thereby protecting mice from mucosal candidiasis [52]. Treatment of mice with three *Lactobacillus* strains that metabolize tryptophan attenuated intestinal inflammation through AHR activation, an effect lost in the presence of an AHR antagonist. *Lactobacillus bulgaricus* OLL1181 was also found to activate the AHR pathway and inhibit colitis in a murine model [52]. It is essential to indicate that the affinities of tryptophan catabolites for AHR differ between mice and humans. Rodent AHR binds to the exogenous ligand 2,3,7,8-tetrachlorodibenzo-p-dioxin (TCDD or Dioxin) with approximately 10-fold higher affinity compared to human AHR, which seems to possess a higher affinity compared to the murine one for various tryptophan-derived ligands [52]. These data reveal that rodent and human AHR exhibit distinct binding selectivity, an important consideration, as predictions about ligand-receptor interactions in humans are often based on rodent studies [52]. In this context, most of the evidence supporting a protective role for AHR activation summarized here derives from murine or in vitro systems; its relevance to humans is inferred largely from interspecies differences in receptor affinity rather than from direct human, specifically athletic, data. In principle, these tryptophan-derived metabolites could attenuate exercise-induced inflammation and support intestinal barrier recovery after intense exercise, thereby integrating tryptophan metabolism with the response to exercise-induced OS. It should be emphasized, however, that direct evidence in athletic populations remains insufficient.

In addition to the mechanisms mentioned, the production of secondary BAs, such as DCA and LCA, appears to have specific actions on health, including inflammation. Notably, it is likely correlated with the appendicular skeletal muscle mass index (ASMI), as alterations in this index are associated with reduced appendicular skeletal muscle mass (ASM) [70]. This is because they interact with metabolic and inflammatory pathways, mainly through FXR and TGR5. FXR-mediated signaling involves both genomic pathways through direct DNA binding and transcriptional regulation, requiring the recruitment of coactivators such as peroxisome proliferator-activated receptor gamma coactivator 1 alpha (PGC-1α) and steroid receptor coactivator 1 (SRC-1) [70]. This nuclear receptor pathway can regulate genes involved in BAs, glucose, and lipid metabolism [70]. Simultaneously, TGR5-mediated signaling may operate through cyclic adenosine monophosphate (cAMP) dependent pathways, leading to protein kinase A (PKA) mediated phosphorylation of cAMP response element-binding protein (CREB) and consequent improvements in energy expenditure and glucose homeostasis [70]. Although direct evidence in athletic populations remains scarce, mechanistic insights from non-athletic contexts are informative. Mancin et al. reported that intestinal dysbiosis in frail elderly or sarcopenic subjects is associated with increased pro-inflammatory bacteria and decreased beneficial microbes, patterns that share mechanistic overlap with dysbiotic shifts observed under excessive training load [55]. Furthermore, subjects with fragility risk and reduced ASM, although preliminarily, appear to exhibit reduced relative abundance of Lactobacilli and *F. prausnitzii*, as well as an increase in the *Bacteroides*/*Prevotella* ratio and potentially pathogenic bacteria, such as *Enterobacteriaceae* [55]. These dynamics can contribute to low-grade chronic systemic inflammation through possible fluctuations in LPS levels and decreases in BAs and SCFAs [71]. SCFAs, through activation of G protein-coupled receptors such as G protein-coupled receptor 41 (GPR41), G protein-coupled receptor 43 (GPR43), and G protein-coupled receptor 109a (GPR109a), and through histone deacetylase (HDAC) inhibition, can also modulate anti-inflammatory processes and reduce OS [71]. All these dynamics contribute to maintaining an optimal health state, and, if out of balance, can induce the onset of diseases that promote, for example, muscle loss and consequently affect physical performance. Additionally, probiotic supplementation may modulate inflammation, thereby reducing pro-inflammatory markers and improving muscle function [28,72].

The gut microbiota, through commensal bacteria, appears to possess direct actions on OS. In particular, *Lactobacilli* and *Bifidobacteria* exhibit antioxidant properties, although the available evidence is still derived from in vitro models [73]. A recent review reported that they exhibit 2,2-diphenyl-1-picrylhydrazyl (DPPH) radical-scavenging activity, cellular antioxidant activity, inhibition of linoleic acid peroxidation, hydroxyl radical scavenging, and reductive capacity [73]. The strains *Levilactobacillus brevis*, *Lactobacillus acidophilus*, and *Bifidobacterium lactis* showed the highest levels of antioxidant activity, while oxygen radical absorbance capacity (ORAC) was found to be a specific characteristic of the *Bifidobacterium longum* CUETM 172 strain [73]. Antioxidant activity seems to be more pronounced in *Lactobacilli*, as they could be facultative anaerobes or microaerophiles, and their antioxidant properties may be determined by thioredoxin and glutathione (GSH)-glutaredoxin systems, and to a lesser extent, SOD and CAT [73]. In *Bifidobacteria*, antioxidant enzymes such as alkyl hydroperoxide reductase C (AhpC), thioredoxin reductase, and Nicotinamide adenine dinucleotide reduced form (NADH) oxidase are more commonly found [73]. The main known mechanisms of antioxidant activity employed by probiotic bacteria to reduce OS in the human organism include regulation of complex signaling networks such as Nuclear factor erythroid 2-related factor 2 (Nrf2) redox signaling, increased levels of antioxidant enzymes, ROS scavenging, metal ion chelation, improvement of intestinal permeability, and modulation of the intestinal microbiota [73]. However, the antioxidant activity of probiotic bacteria in humans remains unclear. These potential mechanisms provide a foundation for examining sport-specific microbial profiles and their practical implications. While specific bacterial strains show antioxidant capacity in vitro and in humans, translating these findings to athletic populations requires careful consideration of dose, timing, and individual baseline microbiota. Moreover, the tryptophan-AHR axis and BAs signaling represent sophisticated mechanisms linking diet, microbiota, and oxidative balance, warranting further investigation in sport-specific contexts. Interindividual variability in AHR polymorphisms and baseline microbiota composition may influence the magnitude of the response, emphasizing the need for personalized approaches based on individual AHR activity and microbial tryptophan-metabolizing capacity (Figure 1).

### 4.2. SCFAs -Mediated Anti-Inflammatory Mechanisms

SCFAs constitute one of the primary mechanisms through which gut microbiota could influence recovery in athletes [41,49,74]. SCFAs, acetate, propionate, and butyrate are metabolites produced by bacterial fermentation of non-digestible dietary fibers within the colon. These compounds account for a substantial portion of the total SCFAs generated daily in the intestine [75,76]. The microbiota primarily produces them in the colon through the fermentation of carbohydrates, known as microbiota-accessible carbohydrates (MACs) [72,76]. Butyrate serves as the primary energy source for colonocytes, supporting epithelial integrity and modulating local inflammation [77]. Acetate and propionate, after being absorbed through the intestinal epithelium, enter the portal circulation and reach the liver. While propionate is largely metabolized in the liver, acetate can be transported into systemic circulation and reach peripheral tissues, such as muscles, the heart, and the brain, and, furthermore, it participates in the regulation of energy metabolism [78]. Consistent with these findings, depletion of the gut microbiota in murine models through antibiotic treatment or a diet low in microbiota-accessible carbohydrates reduced endurance capacity. This effect was reversed by continuous acetate infusion, providing causal, albeit preclinical, evidence that microbiota-derived acetate can sustain exercise capacity [74]. In athletes, SCFAs production is influenced by microbiota composition and dietary fiber intake. A recent review summarized that endurance athletes may exhibit a distinct microbial signature and higher fecal SCFAs levels than sedentary subjects [47]. Barton et al. observed that professional rugby players showed significantly higher levels of fecal acetate, propionate, and butyrate than sedentary controls [13].

Specific microbial signatures and probiotic supplementations may contribute to anti-inflammatory effects in athletes, with SCFAs acting on the nuclear factor kappa-light-chain-enhancer of activated B cells (NF-κB) pathway, a central transcription factor in inflammatory processes [41,71,79,80]. Butyrate, in particular, could act as an HDAC inhibitor, modulating the gene expression of pro-inflammatory cytokines such as IL-1β, IL-6, and TNF-α [81]. This modulation of inflammation is particularly relevant for managing muscle recovery. A balanced gut microbiota and SCFAs could help contain this inflammatory response, facilitating faster and more effective recovery. Scheiman et al. identified *Veillonella atypica* in the microbiota of marathon runners, demonstrating its capacity to metabolize lactate produced during aerobic physical exercise into propionate. This mechanism not only helps convert muscle lactate but also produces SCFAs that can serve as energy substrates [82]. Additionally, the same authors indicated that inoculation of *Veillonella atypica* in murine models improved running performance, suggesting a possible direct mechanism through which specific microbiota signatures could influence recovery and physical performance [82]. However, subsequent studies have not consistently replicated these findings in elite athlete cohorts, reporting no significant differences in *Veillonella atypica* abundance between athletes and sedentary controls, with contrasting results partially attributed to differences in dietary data availability and control, analytical methods, athletes’ training level, and possibly host genetics, in addition to study design [83]. Fontana et al., analyzing 418 publicly available shotgun metagenomic datasets from athletes, moderately active individuals, and sedentary controls, highlighted a significant increase in SCFAs-producing bacteria in athletes compared to sedentary controls, with positive correlations between microbial diversity and SCFAs production levels. In particular, they observed an enrichment of genera such as *Faecalibacterium*, *Eubacterium*, *Blautia*, and *Ruminococcus*, which are known for their capacity to produce SCFAs [48].

SCFAs constitute the most mechanistically characterized microbiota recovery pathway, with butyrate HDAC inhibition and propionate metabolic effects well-documented. However, fecal SCFAs levels may not reflect systemic bioavailability, and interindividual variability in production capacity remains a barrier to standardized interventions (Figure 1).

## 5. Sport-Specific Microbiome Adaptations in Athletes

Different sports disciplines generate distinct selective responses that shape microbiota composition, reflecting their metabolic and physiological demands [84]. Endurance athletes typically develop enhanced populations of *Prevotella*, *Veillonella*, *Faecalibacterium*, and *Roseburia*, likely reflecting the metabolic demands of prolonged aerobic physical exercise [9,82]. This is supported by studies on professional cyclists, which have identified a greater abundance of *Prevotella*, specifically involved in branched-chain amino acid (BCAA) metabolism, relevant for exercise-induced mental fatigue and, although minimally, for protein synthesis [85]. Moreover, marathon runners show increased levels of *Veillonella* following races, which has been shown to metabolize exercise-produced lactate into propionate, potentially reducing muscle fatigue while providing additional energy substrates [82,84]. Strength and power athletes, in contrast, can exhibit an increased abundance of *Bacteroidaceae*, protein-metabolizing bacteria, and specialized pathways for amino acid metabolism, which likely support the anabolic demands of resistance training [84,86,87]. Team-sport athletes instead present hybrid microbiota profiles that combine elements of endurance, strength, and power-sport profiles, given the intermittent nature of team sports [84]. For example, field hockey athletes have been found to have elevated levels of *Lactobacillus acidophilus*, while volleyball athletes exhibit elevated levels of *Ruminococcaceae* [14,84,86].

Training periodization and programming in sport are characterized by the organized variation of volume, intensity, density, frequency, and specificity of training, which induce compositional and functional changes in the gut microbiota that correlate with performance adaptations and recovery status [14,88]. Elite athletes undergoing periodized, programmed training and competition demonstrate phase-specific shifts in microbial enterotype distribution. During the training period, significant changes in microbiota are observed across various disciplines between phases. The preparation phase, compared to the transition phase, is characterized by increased frequency of the *Bacteroides* enterotype and greater abundance of *Bifidobacterium*, *Parabacteroides*, and *Alistipes*, while *Prevotella* decreases. In the specific preparation phase, compared to the general preparation phase, increases in *Blautia* and *Bifidobacterium* are observed, accompanied by decreases in *Bacteroides*. These microbiota changes correlate significantly with variations in aerobic capacity and anaerobic power [88]. In national-level rowing athletes, periods of high training load significantly reduced the *Firmicutes/Bacteroidetes* (F/B) ratio and increased SCFAs concentrations, particularly propionate and butyrate [89]. Similarly, elite volleyball athletes exhibit dynamic gut microbiota stability during training, competition, and recovery, with fluctuations in the F/B ratio that increased during competition and decreased during recovery phases. These athletes also showed significant enrichment of *Rikenellaceae* during rest periods [14]. Overall, these changes correlate with training load, competition metrics, and physiological adaptations, suggesting that microbiota monitoring could serve as a biomarker for training and competition stress and adaptation capacity. It should be noted, however, that the *Firmicutes*/*Bacteroidetes* ratio is increasingly questioned as a reliable standalone biomarker, given its high sensitivity to sequencing methodology, dietary habits, and compositional effects [83]. The informative value of the F/B ratio, therefore, depends on its interpretation alongside genus and species-level composition and functional data, as it retains only a limited, context-dependent value.

While several studies have characterized the microbiota of elite athletes, identifying distinctive patterns associated with recovery and performance, the available evidence remains limited [90]. Nevertheless, preliminary evidence suggests that microbiota diversity differs between sports and during periods of training and competition, likely reflecting physiological stress and recovery capacities. Although these are preliminary results that require further study, understanding these dynamics could optimize the use of periodization strategies that exploit microbiota–host interactions to maximize training adaptations while minimizing the risk of overtraining. Some authors have demonstrated that elite athletes exhibit greater microbial diversity compared to sedentary controls and a lower inflammatory status [12]. Li et al. conducted a multi-cohort study on 543 fecal samples from athletes in three different sports, aerobics, wrestling, and rowing, identifying microbial subgroups associated with different inflammation patterns [91]. In particular, the female rowing subgroup, dominated by *Prevotella*, although not resolved at the species level, was associated with most inflammatory indicators, suggesting a possible risk of intestinal inflammation in some athletes [91]. During intense loading periods, reductions in microbial diversity and increases in potentially pro-inflammatory taxa could be observed [92]. Murtaza et al. studied elite race walkers during a phase of training intensification and observed that dietary patterns influenced microbiota composition, with possible implications for recovery and performance [93].

These findings suggest that microbiota profiles reflect both sport-specific demands and training periodization phases, providing a valuable avenue for monitoring athletic adaptation and recovery processes. Microbiota composition reflects the combined effects of training stimulus and dietary pattern, with distinct signatures emerging across disciplines. Longitudinal changes during periodization suggest potential utility as biomarkers of the balance between training stress and adaptation. However, it is unclear whether the microbiota shapes performance, training shapes the microbiota, or both processes contribute (Table 1).

## 6. Microbiome-Based Recovery Strategies

### 6.1. Nutritional Strategies and Biosynthetic Capacity

Recovery represents a critical component of performance optimization and long-term athletic capacity in elite athletes [1]. Recovery strategies in previous years have focused on physical, nutritional, and pharmacological modalities, but the growing understanding of the role of the gut microbiota in performance has expanded research perspectives on its potential contribution to athletic recovery [9,94]. The relationship between exercise and the gut microbiota is bidirectional, with reciprocal interactions influencing both microbial homeostasis and host physiological adaptations [9,14,15,94,95]. Elite athletes demonstrate gut microbiota resilience characterized by sport-specific compositional and functional adaptations that enhance SCFAs production [14,48,49]. However, intense and prolonged exercise can disrupt this homeostasis by inducing systemic inflammation and increasing ROS production, processes that contribute to muscle damage and may alter gut microbiota composition [9,17,18,59,60]. In this context, the intestinal ecosystem can be influenced not only by these factors but can also modulate them through multiple interaction mechanisms. The gut microbiota can modulate inflammation through the production of SCFAs, which serve as one of the primary mechanisms by which microbiota can influence recovery in athletes by inhibiting the NF-κB pathway and modulating the expression of pro-inflammatory cytokine genes [74,79,80,81].

Beyond the mechanisms described above, microbiota biosynthetic capacity extends to the production of essential micronutrients and metabolic cofactors that may further support athletic recovery. The intestinal ecosystem provides essential nutrients, including vitamins, amino acids, and metabolic cofactors, which can support recovery in elite athletes and potentially create a metabolically advantageous state for regenerative processes [13,96,97,98]. In a re-analysis of 418 publicly available shotgun metagenomic datasets, the enzymatic functional cluster predominant among athletes was positively correlated with a substantially larger set of high-biological-impact synthases (HBIS) than the cluster predominant among sedentary individuals, suggesting a greater microbial biosynthetic potential for health-relevant secondary metabolites [48]. Bacteria belonging to *Firmicutes*, *Actinobacteria*, *Bacteroidetes*, and *Proteobacteria*, such as *Bifidobacterium* and *Lactobacillus*, are able to produce B-complex and K vitamins, as well as transform dietary amino acids into bioactive metabolites that modulate immunity, metabolism, and protein homeostasis, potentially supporting energy metabolism, mitochondrial function, and recovery in elite athletes [97,98,99,100,101]. Barton et al. reported that elite players exhibit enriched metabolic pathways, including amino acid biosynthesis and carbohydrate metabolism, and that these pathways are accompanied by fecal metabolites associated with higher muscle turnover [13]. Although athletic populations generally display enhanced bacterial nutrient-synthesizing capacity, greater enzymatic diversity, and active metabolic pathways, microbiota composition also reflects differences related to diet, training status, and host genetics [9,13,48]. These microbial biosynthetic mechanisms, including SCFAs production and micronutrient synthesis, provide a rationale for targeted interventions to optimize recovery in athletic populations, although further research is required to quantify their precise functional contributions.

### 6.2. Probiotic Interventions and Sleep-Microbiome Axis

Nutritional strategies are a fundamental component for modulating microbiota and optimizing recovery. Adequate dietary fiber intake and diversification of protein sources, along with emphasis on omega-3 (ω-3) fatty acids, appear to optimize gut health in athletic populations [72,94]. Polyphenols, through their prebiotic-like effects, can promote the proliferation of beneficial bacterial genera, including *Akkermansia*, *Lactobacilli*, and *Bifidobacteria*, while undergoing extensive gut metabolism to produce bioactive molecules that modulate inflammation and support recovery [102]. Recent evidence suggests that polyphenol and prebiotic fiber blends have additive beneficial effects on gut microbiota composition [103,104].

Based on these anti-inflammatory mechanisms, several studies have examined the practical application of probiotic interventions to optimize recovery in elite athletes. Previous research has investigated the effects of probiotic supplementation on athletic performance and recovery. Huang et al. reported that *L. plantarum* PS128 supplementation in triathletes improved endurance running performance and increased fecal SCFAs concentrations, without changes in maximal oxygen uptake [105]. A recent two-phase study reported that supplementation with a multi-strain *Lactobacillus* consortium improved sleep quality, energy levels, and intestinal function; the study comprised an open-label phase in the general population and a placebo-controlled phase in an elite soccer team [106]. Furthermore, the effects were associated with modifications in hormonal markers and a reduction in OS and inflammatory markers [106]. A recent review emphasized that probiotics containing *Lactobacilli* and *Bifidobacteria* have been reported to reduce inflammatory markers in athletes, proposing that the immunoregulatory effects of probiotics may be attributed to Treg cell activity and their capacity to enhance IL-10 synthesis, thereby attenuating excessive inflammatory responses [107]. In support of these findings, Maymandinejad et al. recently demonstrated that a multi-strain probiotic formulation comprising *Lactiplantibacillus plantarum* BP06, *Lacticaseibacillus casei* BP07, *Lactobacillus acidophilus* BA05, *Lactobacillus bulgaricus* BD08, *Bifidobacterium infantis* BI04, *Bifidobacterium longum* BL03, *Bifidobacterium breve* BB02, and *Streptococcus thermophilus* BT01, combined with ω-3 supplementation, enhanced sprint swimming performance relative to individual supplementation protocols [108]. Additionally, a six-week intervention study in 22 male cyclists using a combination of *Bifidobacterium longum* CECT 7347, *Lactobacillus casei* CECT 9104, and *Lactobacillus rhamnosus* CECT 8361 produced reductions in OS markers, including 8-hydroxy-2′-deoxyguanosine, malondialdehyde, and oxidized Low-Density Lipoprotein (LDL) cholesterol, compared with baseline [109]. A randomized controlled study involving recreational athletes supplemented with *Lactobacillus plantarum* (10 billion Colony-Forming Units [CFU] per day for 4 weeks) demonstrated improvements in total antioxidant capacity and a reduction in protein carbonyl formation, indicating decreased oxidative damage to proteins [110]. Moreover, they reported improvements in performance on the Yo-Yo intermittent recovery level 1 test, suggesting that enhanced antioxidant protection could translate into measurable athletic benefits [110]. These findings suggest that targeted microbiota interventions may provide measurable benefits against exercise-induced oxidative damage. However, dose–response relationships remain unclear due to heterogeneity in strains, formulations, populations, and intervention durations, as well as limited sample sizes and follow-up. Despite encouraging findings, current evidence remains insufficient to establish standardized, sport-specific probiotic protocols.

Evidence suggests that the combination of sport type and a sport-specific diet is associated with distinct microbiota characteristics, indicating that athletes from different sports disciplines exhibit specific microbial signatures correlated with their dietary and training regimens [111]. To date, we know that probiotic supplementation contributes to optimizing physical performance and health, to managing GI issues and URTIs, especially in aerobic sports contexts compared to anaerobic ones, but it still presents limitations, also due to methodological variability across studies and to its actual effectiveness in sport-specific contexts [28,112]. Probiotics such as *Lactobacilli* and *Bifidobacteria* should generally be recognized as safe (GRAS), but specific considerations should be made for athletes with immune compromise or pre-existing GI problems [113]. Studied adverse events are generally null or mild and transient, including meteorism, abdominal distension, and modifications of bowel habits during the first weeks of treatment, but in the context of sports doping, all supplements in addition to being safe must be verified for the absence of substances prohibited by the World Anti-Doping Agency (WADA) and certification by recognized organizations should be recommended for safe use [114,115,116]. Beyond direct probiotic supplementation, optimizing recovery in elite athletes requires understanding the complex interactions between microbiota composition and sleep regulation, a critical yet often overlooked component of athletic recovery.

Sleep is fundamental to managing athletic performance and recovery, and the gut microbiota is increasingly recognized as capable of influencing sleep through the gut–brain axis [15,80,117,118,119,120]. The gut microbiota, through specific taxa, has been shown to produce and regulate various neurotransmitters that influence sleep [121]. Gamma-aminobutyric acid (GABA) is one of the primary inhibitory neurotransmitters in the central nervous system and can be produced by bacterial species, such as *Lactobacillus* spp. and *Bifidobacterium* spp., through the action of glutamate decarboxylase [121,122]. Similarly, serotonin, a precursor of melatonin, can be influenced by tryptophan metabolism, which is also mediated by microbiota [123,124]. SCFAs, particularly butyrate, can, albeit to a limited extent, cross the blood–brain barrier and potentially influence neurotransmission [125,126,127]. Sleep deprivation and circadian rhythm disruption are associated with significant alterations of gut microbiota [128]. Although derived from clinical insomnia populations rather than athletic cohorts, findings by Li et al. suggest that subjects with acute and chronic insomnia exhibit reduced microbial richness and diversity, depletion of anaerobic and SCFAs-producing bacteria, and an increase in potential pathobionts, patterns that may inform hypothesis-generating research on sleep-microbiota interactions in athletes experiencing sleep disturbances [129]. Athletes, moreover, are exposed to sleep disruptors that are largely absent from clinical insomnia cohorts, notably frequent transmeridian travel with circadian misalignment, irregular competition schedules, and pre-competition psychological stress. These factors could plausibly perturb the sleep-microbiota axis in sport-specific ways, for instance, through cortisol-mediated changes in gut permeability and microbial composition. This, however, remains a hypothesis-generating avenue, as direct evidence in athletes remains insufficient.

These sleep-microbiota interactions, combined with the inflammatory and metabolic mechanisms described previously, suggest potential applications for targeted probiotic interventions in athletic populations. This integrated approach, combining SCFAs-mediated anti-inflammatory pathways, targeted probiotic interventions, and optimization of the sleep-microbiota axis, represents a possible comprehensive strategy for enhancing recovery processes in elite athletes (Table 2).

## 7. Strengths and Limitations

Several studies support the potential use of microbiota-based approaches for athletic recovery [84,130]. Mechanistic pathways are biologically plausible, with well-characterized SCFAs effects, including butyrate-mediated HDAC inhibition and propionate production from exercise-derived lactate via *Veillonella* metabolism, although this mechanism awaits consistent replication in elite athlete cohorts. Tryptophan metabolites interact with specific receptors via AHR activation, whereas BAs target established molecular pathways, including FXR and TGR5. The anti-inflammatory effects of *Lactobacillus* and *Bifidobacterium* supplementation have been reported across multiple randomized controlled trials, although heterogeneity in strains, dosages, and study designs limits direct comparisons. Interventional studies have reported measurable effects in objective parameters, including reductions in inflammatory markers, improvements in performance tests, and enhancements in sleep quality. These findings progress logically from a mechanistic understanding to practical applications, supporting a translational approach to optimizing microbiota-based recovery.

Sample sizes in intervention studies typically involve fewer than 50 participants. Methodological approaches vary considerably across studies, with different analytical techniques, probiotic formulations, dosages, and intervention durations, limiting direct comparisons between trials [131]. Cross-sectional designs predominate over longitudinal designs, limiting causal inference regarding the relationship between microbiota composition and recovery outcomes. Athletic populations present unique challenges in controlling confounding variables, including diet, training periodization, stress levels, and antibiotic exposure, all of which significantly influence microbiota composition. Consequently, the specificity of athletic populations requires careful consideration when extrapolating findings across different athletic disciplines and training contexts, although this constitutes an important research direction. Beyond these methodological limitations, publication bias may also affect the available literature, particularly given the small sample sizes and industry funding reported in some trials. Moreover, a persistent translational gap remains, as much of the mechanistic evidence derives from in vitro or animal studies and requires validation in humans, particularly in elite athletes under competitive conditions.

Interindividual variability in the response to probiotics remains poorly characterized. The baseline gut microbiota composition, host genetics, dietary patterns, and antibiotic exposure all profoundly influence colonization and efficacy. Current group-level analyses may obscure responder versus non-responder phenotypes, which can explain the heterogeneous trial outcomes. Predictive biomarkers enabling personalized strain selection are critically needed.

## 8. Practical Applications

The implementation of microbiota analysis protocols in sports necessitates specific considerations regarding sampling timing, analytical methodologies, and the interpretation of results within the athletic context. Mancin et al. recently proposed guidelines for standardizing microbiome analysis in sports, emphasizing the importance of standardized protocols for sample collection, preservation, and analysis [131]. 16S rRNA sequencing can provide information on microbial composition at the phylum, family, and genus levels, while Shotgun Metagenomic approaches allow for more detailed functional characterization at the species and strain levels [131]. Integration with metabolomic analyses of SCFAs could provide additional functional information on active metabolic pathways. Combining microbiota data with traditional hormonal markers of muscle recovery could provide a more comprehensive assessment of the athlete’s recovery status and serve as early indicators of overreaching or overtraining syndrome [132,133]. Bacterial biomarkers such as *Faecalibacterium prausnitzii* and *Escherichia coli* have been investigated in inflammatory bowel disease (IBD), colorectal cancer (CRC), and other gastrointestinal disorders, including irritable bowel syndrome (IBS). As a hypothesis for future research rather than a current recommendation, these could inform the development of analogous biomarkers in sports medicine. In athletic populations, the species *Faecalibacterium prausnitzii*, *Roseburia hominis*, and *Akkermansia muciniphila* have been associated with potentially protective properties [134,135]. However, the functional relevance of these findings for performance, recovery, or training adaptation remains unclear, as these biomarkers are currently validated for GI disease assessment rather than athletic monitoring, and their translational application in sports medicine remains to be established.

Probiotic prescription for recovery, despite still not being fully mature, should consider strain specificity, appropriate dosages, administration timing, and periods of intense training and competition. Current evidence suggests that probiotic dosages typically range from 10^9^ to 10^11^ CFU daily, and that intervention durations of 2 to 12 weeks produce measurable outcomes in athletic populations in small-scale trials [28,106,107,108,109,110,136,137,138]. However, the optimal dosage, strain combination, and timing remain undefined, and no standardized protocol has been validated in large-scale athletic populations. These figures should therefore be regarded as the ranges tested to date rather than as evidence-based targets, and their implementation remains, at present, an experimental and necessarily individualized approach. Sport-specific timing and individual factors may influence efficacy, suggesting that personalized approaches could be beneficial but require further investigation. Response to probiotic interventions varies between athletes [112,131]. Key predictive factors include baseline gut microbiota composition, dietary fiber intake, host genetic polymorphisms in immune receptors, and recent antibiotic exposure [9,48,50,72,131]. Integrating these factors through baseline microbiota profiling could support personalized strain selection, though this approach requires validation in larger cohorts to improve intervention efficacy and reduce trial heterogeneity. Athletes with recurrent URTI or persistent exercise-induced GI symptoms represent priority candidates for probiotic intervention [28].

Beyond identifying suitable candidates, safe and effective implementation requires careful selection of the product. Probiotics used in athletic populations should have well-documented, regulated safety profiles and GRAS status. Athletes should ensure product traceability and verify each production batch against the current WADA Prohibited List. Practically, well-documented safety should entail strain-level characterization, evidence from controlled clinical trials, and post-market surveillance, with verification responsibilities shared between the athlete’s medical staff and national anti-doping organizations [113,114,115,116]. The variability in probiotic supplement quality necessitates particular attention in product selection, with critical factors including the viable count of microorganisms at the time of consumption, product stability during storage, the absence of microbial contaminants, and the specificity of the strains used.

Nutritional optimization for microbiota and recovery involves increasing daily prebiotic fiber intake, diversifying protein sources, incorporating natural fermented foods, and limiting ultra-processed foods [9,72]. Increasing intake of fermentable dietary fiber supports both SCFAs production and tryptophan-derived metabolite signaling [50,51,139]. Successful implementation should integrate sleep hygiene, psychological and physical stress management, proper hormonal regulation, periodization of training and nutrition, regular mealtimes, and exposure to natural light [80,137,140,141,142]. This is supported by recent evidence indicating that the gut microbiota responds dynamically to training periodization, suggesting that nutritional strategies should be adjusted across different training phases to optimize both performance and recovery adaptations [84,88,89].

**Figure 2:**
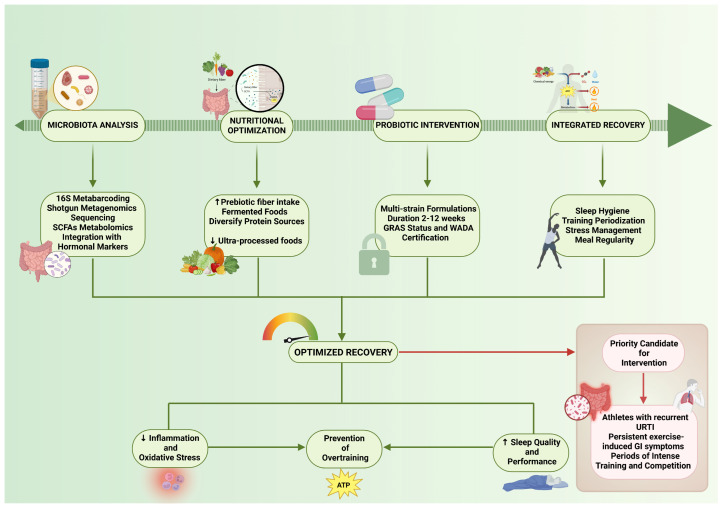
Microbiome-based recovery optimization: practical applications in elite athletes. (Applications complement established recovery strategies). (↑ = Increased; ↓ = Decreased).

The integration of microbiota assessment with traditional recovery markers is a promising avenue for optimizing athletic recovery; however, further research is needed to establish standardized protocols (Figure 2).

## 9. Future Directions

The integration of metagenomic, metabolomic, and proteomic data could provide a more comprehensive understanding of microbiota–host interactions in the context of athletic recovery [143,144]. Artificial intelligence (AI) and machine-learning approaches are increasingly proposed for this purpose, as pattern-recognition and predictive models are better suited to high-dimensional, longitudinal multi-omics data than conventional statistics. The use of predictive models to analyze these complex datasets represents a promising frontier. The development of technologies for real-time monitoring of the microbiota and its metabolites provides a valuable approach to recovery in athletes. For example, the development of portable sensors could allow rapid adjustments of recovery strategies based on the athlete’s microbial status. Furthermore, integrating host hormonal data with microbiota data could enable personalized, targeted treatment approaches. Different sports could require specific microbiome optimization strategies for recovery. Endurance or strength sports could benefit from differentiated approaches based on specific metabolic and physiological demands. The development of precision probiotics, postbiotics, or synbiotics engineered specifically for athletes offers a novel approach to enhance SCFAs production or modulate immune responses during recovery. Alternatively, administering purified SCFAs or postbiotic metabolites could offer more standardized and predictable interventions, thereby avoiding the variability associated with live probiotic formulations.

Realizing this potential requires confronting several barriers. Sequencing and metabolomic profiling remain costly and slow relative to the decision-making timescales of applied sport. Best-practice frameworks for sampling, storage, and analysis have been proposed, and 16S rRNA Metabarcoding and Shotgun Metagenomics have been critically appraised in athletic cohorts, but their uptake remains uneven [131,145]. Comparability is therefore limited less by the absence of standards than by their inconsistent adoption, and much of the existing corpus cannot be harmonized retrospectively. Marked interindividual variability, in baseline composition and in response to intervention, restricts the generalizability of any single recommendation. Interpreting microbiota data in a performance context requires expertise seldom available within a performance department, and adherence to dietary or supplementation regimens is difficult to sustain across a congested competitive season.

The priorities for the coming five to ten years follow from this. Most pressing are adequately powered longitudinal and, where feasible, randomized studies in well-characterized elite cohorts, conducted under the harmonized protocols already available. The present evidence base is small, heterogeneous, and largely cross-sectional, and cannot support causal inference. Descriptive and comparative studies have been essential in establishing that the athlete’s gut microbiota is distinguishable from that of sedentary populations, and longitudinal designs in particular have begun to reveal how it responds to training load and competition. Building on this foundation, the field is now well placed to extend from taxonomic description toward functional characterization.

A related objective is a curated, open-access athlete microbiome databank, aggregating taxonomic, functional, and phenotypic data under harmonized metadata standards and, where possible, coupled to a biobank of matched specimens. Such a repository would permit meta-analyses across cohorts that no single laboratory could assemble, and would yield the sport, sex, and competitive level-specific reference ranges whose absence is itself a principal obstacle to interpretation [131,145]. On this foundation, predictive biomarkers of individual response could be validated, allowing responder stratification and personalized strain selection. Live probiotics could be compared head-to-head with postbiotics and purified metabolites in controlled trials, and mechanistic work could be conducted in athletes rather than extrapolated from preclinical models.

The risks, finally, warrant explicit consideration but are seldom addressed. Perturbing an already stable, sport-adapted community may prove counterproductive. The long-term consequences of repeated or high-dose probiotic supplementation in athletes are unknown, as are the interactions between probiotics, other supplements, and the medications used in elite sport. Aggregating athlete microbiota data is scientifically necessary but raises its own difficulties, since such data are at once personally identifying and commercially sensitive. Consent, ownership, and governance should therefore be settled at the design stage rather than retrospectively. More broadly, the prospect of microbiota-targeted enhancement, including microbiota manipulation pursued as a form of biological doping, warrants anticipatory rather than reactive attention from the sports medicine and anti-doping communities.

**Figure 3:**
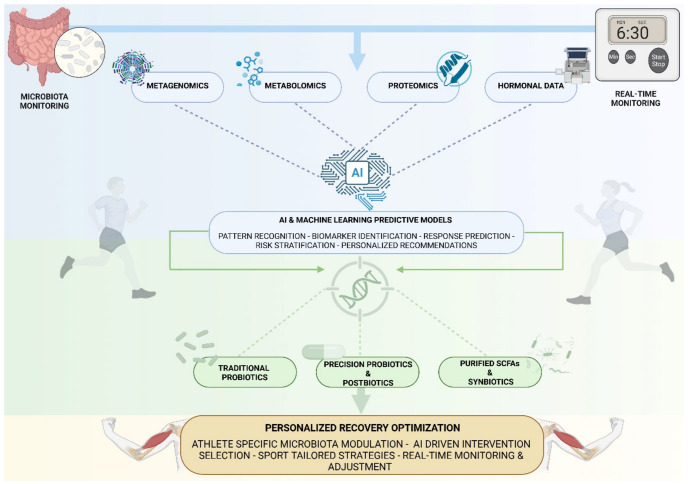
Integration of Artificial Intelligence, Multi-Omics, and Real-Time Monitoring for Personalized Microbiome-Based Recovery in Elite Athletes. (The framework outlines a prospective research direction). (AI = Artificial Intelligence).

The future of microbiota-based recovery in elite sport may lie in individualized, data-informed systems that integrate multi-omics profiling, real-time monitoring, and precision nutritional interventions, potentially moving sports medicine toward more personalized recovery protocols (Figure 3).

## 10. Conclusions

Emerging evidence suggests that the gut microbiota may contribute meaningfully to recovery in elite athletes. The proposed mechanisms by which the microbiome may influence recovery are multiple and interconnected, including the production of SCFAs with anti-inflammatory properties, modulation of the gut–brain axis to improve sleep, management of OS, optimization of immune function, and hormonal regulation. Candidate applications, which remain experimental, include the use of selected probiotic strains, nutritional strategies to promote SCFAs production, and the integration of microbiota assessments with hormonal markers in athlete monitoring; postbiotic approaches currently remain a prospective research direction rather than a practical option. Nutrition remains the primary modulating factor of the gut microbiota, and current evidence suggests that nutritional strategies should be adapted to training and competition phases, although this approach requires prospective validation in elite athletes [84,146]. Despite these advances, significant research gaps remain regarding standardized analytical frameworks, appropriate statistical approaches for high-dimensional microbiome data, intervention protocols, predictive biomarkers of individual responses, personalized strategies based on host genetics and sport specificity, and the effectiveness of long-term interventions [145,147]. Future research must address these limitations to establish evidence-based guidelines.

Microbiota-based strategies for recovery are gaining increasing attention in sports medicine, providing new opportunities to optimize recovery and performance through the modulation of intestinal health. Despite a compelling mechanistic rationale, however, current evidence remains limited and heterogeneous, particularly in elite athletes. Microbiota assessment should therefore complement, rather than replace, established recovery strategies. Multidisciplinary collaboration among coaches, strength and conditioning specialists, sports nutritionists, and sports medicine physicians will be essential to translate emerging evidence into practice. Further mechanistic insights into how the gut microbiota shapes recovery may ultimately support more precise, individualized interventions in sports medicine.

## Figures and Tables

**Table 1:** Sport-Specific and Periodization-Related Gut Microbiota Signatures in Elite Athletes. (Associations are cross-sectional, based on small sample sizes, and context dependent. Causality and the specificity of these signatures to individual sports remains unestablished).

Sport/Training Phase	Microbial Taxa Changes	Functional Implications	References
Endurance athletes	↑ *Prevotella*, *Veillonella*, *Faecalibacterium*, *Roseburia*	BCAA metabolism; Lactate to Propionate conversion (preliminary data); ↓ Muscle fatigue	Mohr 2020 [9]; Scheiman 2019 [82]; Petersen 2017 [85]
Strength and Power athletes	↑ *Bacteroidaceae*; Protein-metabolizing bacteria; Specialized amino acid metabolism pathways	Support for the anabolic demands of resistance training	O’Donovan 2020 [86]; Kuibida 2023 [87]
Team Sport athletes	↑ *Lactobacillus acidophilus*; ↑ *Ruminococcaceae*	Hybrid metabolic profile supporting intermittent high-intensity efforts	Carlone 2025 [14]; Carlone 2025 [84]; O’Donovan 2020 [86]
Preparation vs. Transition phase	↑ *Bacteroides* enterotype, *Bifidobacterium*, *Parabacteroides*, *Alistipes*; ↓ *Prevotella*	Correlated with variations in aerobic capacity (VO_2_max)	Akazawa 2023 [88]
Specific vs. General preparation phase	↑ *Blautia*, *Bifidobacterium;* ↓ *Bacteroides*	Correlation with aerobic and anaerobic power adaptations	Akazawa 2023 [88]
High training load periods	↓ *Firmicutes/Bacteroidetes* ratio; ↑ SCFAs (Propionate, Butyrate)	Increased production of anti-inflammatory and energy-yielding metabolites	Charlesson 2025 [89]
Competition vs. Recovery Phases	↑ *Firmicutes/Bacteroidetes* ratio (Competition periods); ↑ *Rikenellaceae* (Rest periods) ↓ *Firmicutes/Bacteroidetes* ratio (Recovery periods)	Potential biomarker for training stress and adaptation; Dynamic stability during periodization	Carlone 2025 [14]

(↑ = Increased; ↓ = Decreased; BCAA = Branched-Chain Amino Acids; VO_2_max = Maximal Oxygen Consumption; SCFAs = Short-Chain Fatty Acids).

**Table 2:** Probiotic interventions for optimizing recovery in athletes. (Studies differ in strain, dosage, duration, outcomes, and population).

Probiotic Strain(s)	Population	Dosage	Duration	Main Outcomes	References
*Lactobacillus plantarum* PS128	High-level Triathletes	3 × 10^10^ CFU/Day	4 Weeks	↑ Endurance performance; ↑ Fecal SCFAs (acetic, propionic, and butyric acid)	Huang 2020 [105]
Multi-strain *Lactobacillus* consortium (*L. acidophilus* FB0012, *L. plantarum* FB0015, and *L. rhamnosus* FB0047)	Elite Soccer Players	1 × 10^10^ CFU/Day	2 Weeks	↑ Sleep quality, Energy levels, Intestinal function, Free Testosterone/Cortisol ratio; ↓ Oxidative stress markers	Bongiovanni 2025 [106]
*Lactiplantibacillus plantarum* BP06, *Lacticaseibacillus casei* BP07, *L. acidophilus* BA05, *L. bulgaricus* BD08, *Bifidobacterium infantis* BI04, *B. longum* BL03, *B. breve* BB02, *Streptococcus thermophilus* BT01 + ω-3	High-level Swimmers	4.5 × 10^11^ CFU/Day	8 Weeks	↑ Sprint swimming performance	Maymandinejad 2025 [108]
*Bifidobacterium longum* CECT 7347, *Lactobacillus casei* CECT 9104, *L. rhamnosus* CECT 8361	Amateur Cyclists	1 × 10^9^ CFU/Day	6 Weeks	↓ 8-Hydroxy-2′-deoxyguanosine, Malondialdehyde, Oxidized LDL cholesterol	Sánchez Macarro 2021 [109]
*Lactobacillus plantarum*	Recreational Athletes	1 × 10^10^ CFU/Day	4 Weeks	↑ Total antioxidant capacity, Yo-Yo Intermittent Recovery Level 1 test performance; ↓ Protein carbonyl formation	Santibañez-Gutierrez 2025 [110]

(↑ = Increased; ↓ = Decreased; CFU = Colony-Forming Units; ω-3 = Omega 3; LDL = Low-Density Lipoprotein; Yo-Yo IR1 = Yo-Yo Intermittent Recovery Level 1 test).

## Data Availability

The original contributions presented in this study are included in the article/Supplementary Materials. Further inquiries can be directed to the corresponding author.

## Supplementary Materials

The following supporting information can be downloaded at: https://www.mdpi.com/article/10.3390/nu18142403/s1, File S1: Scale for the Assessment of Narrative Review Articles—SANRA.
